# Sneaking Out for Happy Hour: Yeast-Based Approaches to Explore and Modulate Immune Response and Immune Evasion

**DOI:** 10.3390/genes10090667

**Published:** 2019-08-31

**Authors:** Gaëlle Angrand, Alicia Quillévéré, Nadège Loaëc, Chrysoula Daskalogianni, Anton Granzhan, Marie-Paule Teulade-Fichou, Robin Fahraeus, Rodrigo Prado Martins, Marc Blondel

**Affiliations:** 1 University Brest, Inserm, EFS, UMR 1078, GGB, F-29200 Brest, France; 2 University Paris 7, Inserm, UMR, 1162 Paris, France; 3 Chemistry, Modelling and Imaging for Biology, CNRS UMR9187, Inserm U1196, Institut Curie, University Paris-Sud, University Paris Saclay, F-91405 Orsay, France; 4 Infectiologie et Santé Publique, INRA, University de Tours, UMR1282 Nouzilly, France

**Keywords:** yeast, *Saccharomyces cerevisiae*, immune system, immune evasion, adjuvant for vaccines, yeast cell wall, G-quadruplexes (G4), G4 ligands, nucleolin (NCL), Epstein–Barr virus (EBV), oncogenic viruses

## Abstract

Many pathogens (virus, bacteria, fungi, or parasites) have developed a wide variety of mechanisms to evade their host immune system. The budding yeast *Saccharomyces cerevisiae* has successfully been used to decipher some of these immune evasion strategies. This includes the *cis*-acting mechanism that limits the expression of the oncogenic Epstein–Barr virus (EBV)-encoded EBNA1 and thus of antigenic peptides derived from this essential but highly antigenic viral protein. Studies based on budding yeast have also revealed the molecular bases of epigenetic switching or recombination underlying the silencing of all except one members of extended families of genes that encode closely related and highly antigenic surface proteins. This mechanism is exploited by several parasites (that include pathogens such as *Plasmodium*, *Trypanosoma*, *Candida*, or *Pneumocystis*) to alternate their surface antigens, thereby evading the immune system. Yeast can itself be a pathogen, and pathogenic fungi such as *Candida albicans*, which is phylogenetically very close to *S. cerevisiae*, have developed stealthiness strategies that include changes in their cell wall composition, or epitope-masking, to control production or exposure of highly antigenic but essential polysaccharides in their cell wall. Finally, due to the high antigenicity of its cell wall, yeast has been opportunistically exploited to create adjuvants and vectors for vaccination.

## 1. Yeast as a Popular Model for Basic Research and Chemical Genetics Approaches

**Figure 1:**
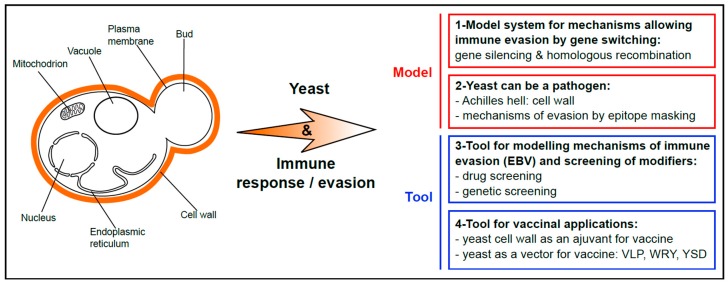
Scheme summarizing the various use of yeast to explore and modulate immune response and immune evasion. Yeasts are fungi and, as such, possess a cell wall that surrounds their plasma membrane, in addition to the typical features of eukaryotic cells (that include existence of a nucleus, mitochondria, vacuoles which are hydrolytic organelles similar to the lysosomes in higher eukaryotes, endoplasmic reticulum etc.). 1. Yeast is a model system for mechanisms based on gene repression and switching that allow immune evasion of many parasites. 2. Yeast can also be a pathogen, notably for humans and animals. Its cell wall is highly antigenic, and yeast has developed various mechanisms of epitope-masking to evade the host immune system. 3. Yeast is a tool for modelling the mechanism that allows immune evasion of the Epstein–Barr virus (EBV). This system has been successfully used to isolate drugs interfering with EBV stealthiness and to identify host cell factors involved in immune evasion of this oncovirus. 4. The antigenicity of the yeast cell wall has been biotechnologically exploited as an adjuvant for vaccination. In addition, yeast cells, heat-killed or alive, have been directly used as vectors for various antigens, hence combining the vaccine and the adjuvant in the same particle (VLP, virus-like particle; WRY, whole recombinant yeast; YSD, yeast surface display).

For many decades, yeast has been a popular and powerful model to study basic cell biology mechanisms that include DNA replication [1] and transcription [2], cell cycle regulation [3], vesicular transport [4], autophagy [5], and cell death [6]. This is nicely illustrated by the numerous yeast scientists in the list of Nobel Prize laureates, in particular in Medicine and Physiology [7]. For about 20 years, yeast has also been exploited as a tool for chemical genetics approaches, in particular in the field of humans and animal disorders. Hence, yeast-based approaches to model and exploit physiopathological mechanisms responsible for several human diseases have been successfully developed. The most evident situations are when the key physiopathological factors are functionally conserved from yeast to humans, for example in the field of inherited mitochondrial disorders [8,9]. However, yeast models based on the expression of key players that do not exist in yeast are also possible, as for example yeast models for Huntington or Parkinson diseases ([10,11,12]; for review: [13]). Altogether, numerous yeast-based models for human disorders have been developed and successfully used for both the identification of candidate drugs and chemical probes (e.g., yeast model for prion-based diseases [14,15]), or of new physiopathological actors and thus of new intervention points and therapeutic targets (e.g., yeast model for Huntington disease [10]). As for viruses and parasites, in particular those affecting humans, some yeast-based models were also instrumental for deciphering various aspects of their biology [16], including, rather unexpectedly, their ability to evade the immune system [17]. In addition, yeast can also be a pathogen, and pathogenic fungi such as *Candida* represent a serious threat to human health. One of the Achilles heel of these pathogenic yeasts is their cell wall, which is at the same time essential for yeast growth and highly antigenic. Hence, as most if not all the pathogens, pathogenic yeasts have developed various strategies to evade the host immune system. Finally, the high antigenicity of yeast cells, due to their cell wall, has been exploited as novel alternatives for the development of vaccines. All these aspects of the link between yeast and immune system modulation are the topic of this review article and are summarized in Figure 1: let us sneak out for happy hour.

## 2. The Long-Lasting Love Affair between EBV and the Budding Yeast *Saccharomyces cerevisiae*

The Epstein–Barr gammaherpesvirus (EBV) is the first oncogenic virus discovered in humans [18,19,20]. Indeed, EBV has been known for more than 50 years to be tightly associated with certain human malignancies and, as of today, EBV is still one of the most effective means for transforming and immortalizing human B-cells. More than 90% of the human population worldwide is infected by EBV whose primary infection remains asymptomatic most of the time but, when it occurs in teenagers and adults, it can be responsible for the infectious mononucleosis, also known as “kissing disease” as the virus is spread through saliva. Mononucleosis is a self-limiting lymphoproliferative disorder that in some cases can be quite harmful. Like all gammaherpesviruses, EBV is latent thanks to its ability to evade the host immune system, thus persisting in most infected people as a lifelong asymptomatic infection [21]. However, it can be responsible for severe lymphoproliferative disorders, notably in patients suffering some forms of immune suppression such as grafted patients receiving immunosuppressive treatments, or human immunodeficiency virus (HIV)-infected people. EBV has also been associated, most of the type in combination with a co-factor, to various cancers that include the Burkitt and Hodgkin lymphomas, nasopharyngeal carcinoma and 10% of gastric cancers [22]. Altogether, more than 1% of cancers are associated with EBV, as at least 200,000 new cases of EBV-associated cancers are estimated per year worldwide [23], as compared to an estimated total of 18 million cancers. For all these reasons, various aspects of EBV biological cycle, including the regulation of its genome replication and maintenance, induction of its lytic cycle, or mechanisms at the basis of its ability to evade the immune system, that are related to its tumorogenicity have been the subject of extensive studies. For all these aspects of EBV biology, the use of the budding yeast *S. cerevisiae* model has been instrumental for more than 30 years and fruitfully contributed to decipher some key aspects of EBV life cycle. This has been recently presented in a review article [24] and includes the identification of (i) autonomous replication sequences (ARS) in the circular double strand DNA episome of EBV [25], (ii) genes encoding human proteins required for EBV genome maintenance [26] and, (iii) the interplay of these host factors with the virally encoded Epstein-Barr nuclear antigen 1 (EBNA1) protein which is the genome maintenance protein of EBV [27,28]. In addition, the transcriptional activity of the BamHI Z Epstein-Barr virus replication activator (ZEBRA), an EBV-encoded transcription factor at the top of a cascade event leading to expression of EBV lytic cycle genes [22], has been recapitulated in budding yeast, allowing the identification of the regions of ZEBRA crucial for its transcription factor activity as well as two natural EBV promoters that are activated by ZEBRA, which include the promoter of ZEBRA itself [29,30]. Budding yeast has also been instrumental to study the oncogenic activity of EBV, in particular to show that BGLF4, the EBV representative of the CHPK (conserved herpesvirus-encoded protein kinase), displays a CDK (cyclin-dependent kinase)-like activity. This has been shown by showing that BGLF4 is able to complement, in yeast, the cell cycle arrest induced by defects in Cdk1/Cdc28, the main yeast CDK that controls the progression through all the phases of the budding yeast cell cycle and which is the homolog of CDK1/CDC2 in humans [31]. This CDK-like activity of BGLF4 most probably contributes to the ability of EBV to promote tumor formation by inducing the phosphorylation of the retinoblastoma (Rb) tumor suppressor gene and of the lamin A/C, two events normally catalyzed by human CDKs and that are necessary for progression into the cell cycle. In support of this model is the observation that Rb is properly phosphorylated in cells infected by EBV [32]. Finally, more recently, budding yeast has been successfully used to investigate the mechanisms at the basis of the immune evasion of EBV. This will be the subject of the following section.

## 3. Use of Budding Yeast to Model and Decipher the Mechanisms at the Basis of EBV Stealthiness

**Figure 2:**
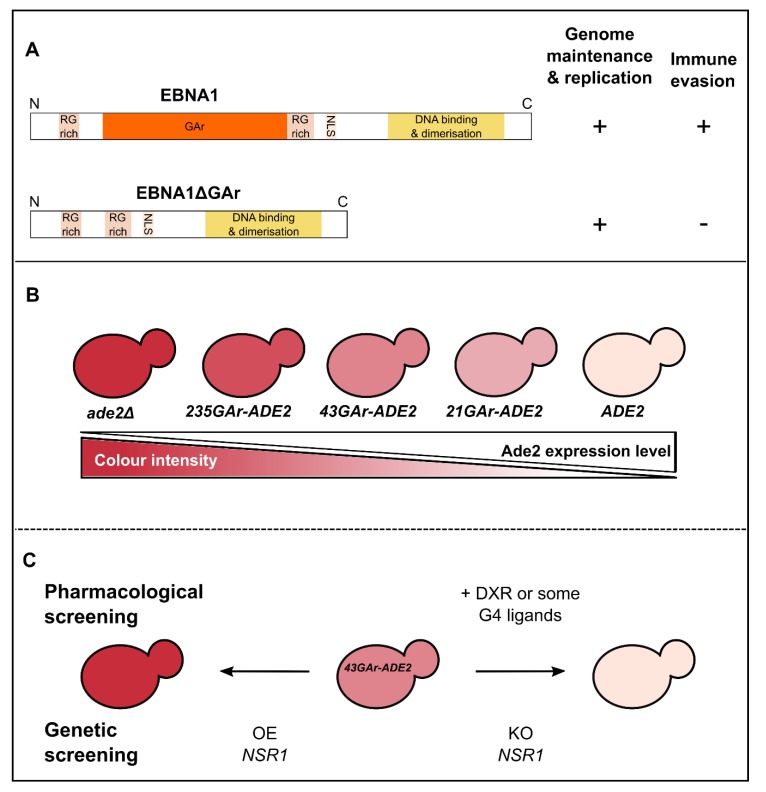
Rationale of the yeast-based assay for immune evasion of EBNA1/EBV. (**A**) Schematic representation of EBNA1 and EBNA1ΔGAr proteins that highlights the crucial role of the central GAr (glycine-alanine repeats) domain for immune evasion: EBNA1ΔGAr is still able to fulfil its essential functions in viral genome maintenance and replication but does not evade the immune system. (**B**) Principle of the *ADE2* reporter gene-based assay. Yeast cells that express a normal level of Ade2p (the product of the *ADE2* gene) form white colonies whereas cells that do not express Ade2p (*ade2Δ* cells) form red colonies due to the accumulation and consecutive oxidation of a by-product of the adenine biosynthesis pathway. Any intermediate level of Ade2 leads to pink colonies whose color intensity is inversely proportional to the Ade2p level. GAr domains of various lengths were fused to the *ADE2* gene. As for EBNA1 in human cells, GAr inhibits the translation of its own mRNA in yeast. Please note that this effect is length-dependent as in human cells, as the longest GAr tested (235GAr) leads to a strong inhibition of translation, giving rise to dark pink colonies, whereas the shortest GAr (21GAr) gives light pink colonies. For screening purpose, the 43GAr, which gives pink colonies, has been used. (**C**) Principle of the pharmacological and drug screenings based on the *43GAr-ADE2* yeast strain. Drugs able to interfere with GAr ability to inhibit translation, such as doxorubicin (DXR) [39] or various G-quadruplex ligands [41,54] lead to whiter colonies that express more 43GAr-Ade2p. In contrast, the overexpression (OE) of host cell genes involved in the GAr inhibitory effect on translation, such as *NSR1* that encodes the yeast nucleolin (NCL in humans), leads to redder colonies as the inhibition of *43GAr-ADE2* expression is exacerbated. Finally, the knockout (KO) of the *NSR1* gene relieves the inhibitory effect of GAr, leading to white colonies.

As stated above, EBV is a latent virus that evades the host immune system but, fortunately, it has an Achilles heel: its aforementioned genome maintenance protein EBNA1. Indeed, due to its crucial role in both EBV genome replication and maintenance, EBNA1 must be expressed in all EBV-infected cells or the virus would be lost upon host cell division [27,28,33]. On the other hand, EBNA1 is highly antigenic and cytotoxic CD8^+^ T cells directed towards EBNA1 epitope are present in every EBV-infected individuals. Hence, EBV evolved a strategy to limit the expression of EBNA1 to the minimal level to fulfil its essential function in replication and maintenance of the viral genome and, at the same time, to minimize its detection by the immune system. This stealthiness strategy is based on the ability of EBNA1 to limit in *cis* the translation of its own mRNA and involves the central glycine-alanine repeat (GAr) of EBNA1 [34,35]. The GAr is dispensable for the essential functions of EBNA1 in EBV genome replication and maintenance, but is critical for EBNA1 stealthiness as an EBNA1 that lacks the GAr (EBNA1ΔGAr) is unable to evade the immune system (Figure 2A) [34,35,36,37]. Deciphering this mechanism is the goal of several teams as it is commonly considered to be a therapeutic target to unveil EBV-infected cells, including tumor cells from EBV-related cancers, to the immune system. Hence there was a clear need for a convenient eukaryotic cell-based assay that would allow performance of high-throughput screenings that include both drug screening (to isolate chemical probes that would serve as tool to study this mechanism of stealthiness and also candidate drugs to unveil EBV-related cancers to the immune system) and genetic screening (to isolate host cell factors involved in this process and that may therefore serve as original therapeutic targets). Indirect evidence from the literature ([38] reviewed in [24]) suggested that budding yeast might be a good option as GAr-based inhibition of translation in *cis* appears to be operant in this model organism. These preliminary observations also suggested that the mechanism of GAr-based inhibition of translation, which is at the basis of immune evasion of EBNA1, and thus of EBV, is conserved from yeast to humans. For all these reasons, a yeast model that recapitulates all the aspects of GAr-based inhibition of translation, including GAr length dependency, has been developed [17,39]. This model is based on the use of the *ADE2* reporter gene [40]. Indeed, this yeast gene encodes Ade2p, one of the enzymes of the adenine biosynthesis pathway which, when absent, leads to red color of yeast cells grown on rich medium (hence supplemented in adenine) due to accumulation of the otherwise cell-limited phosphoribosylaminoimidazole, a metabolic by-product of the adenine biosynthesis pathway, which appears red upon oxidation. In contrast, when expressed at a sufficient level, the *ADE2* gene leads to white yeast cells whereas any intermediate level of expression leads to a pink color whose intensity is inversely proportional to the expression of Ade2p (Figure 2B). This reporter gene has also been chosen because it encodes a protein that is very stable, as EBNA1. Then, sequences encoding GAr domains of different lengths have been fused to the *ADE2* reporter gene. As in human cells, this led to a GAr length-dependent *cis* inhibition of the translation of *GAr-ADE2* mRNA which is shown by a gradient of pink color of yeast colonies: a short GAr (21GAr; 21 residues) only slightly affects the expression of Ade2p, thereby leading to light pink colonies, a 43GAr leads to medium pink colonies whereas yeast cells expressing a full-length *GAr-ADE2* fusion (235GAr; 235 residues) as a sole source of Ade2p form dark pink colonies (Figure 2B). Of note, similarly to situation in human cells, GAr does not affect transcription nor mRNA or protein stability. Hence, it specifically affects translation, in *cis* and in a length-dependent manner. This represented a first validation of the yeast model and further suggested that, should host cell factors involved in GAr-based inhibition of translation exist, then they are conserved from yeast to humans.

The yeast model was further validated since it allowed the identification drugs that specifically interfere with the GAr-based limitation of translation both in yeast and human cells, and that later on turned to be also able to interfere specifically with the GAr-based limitation of antigen presentation by the major histocompatibility complex (MHC) class I (Figure 2C, top) [39].

**Figure 3:**
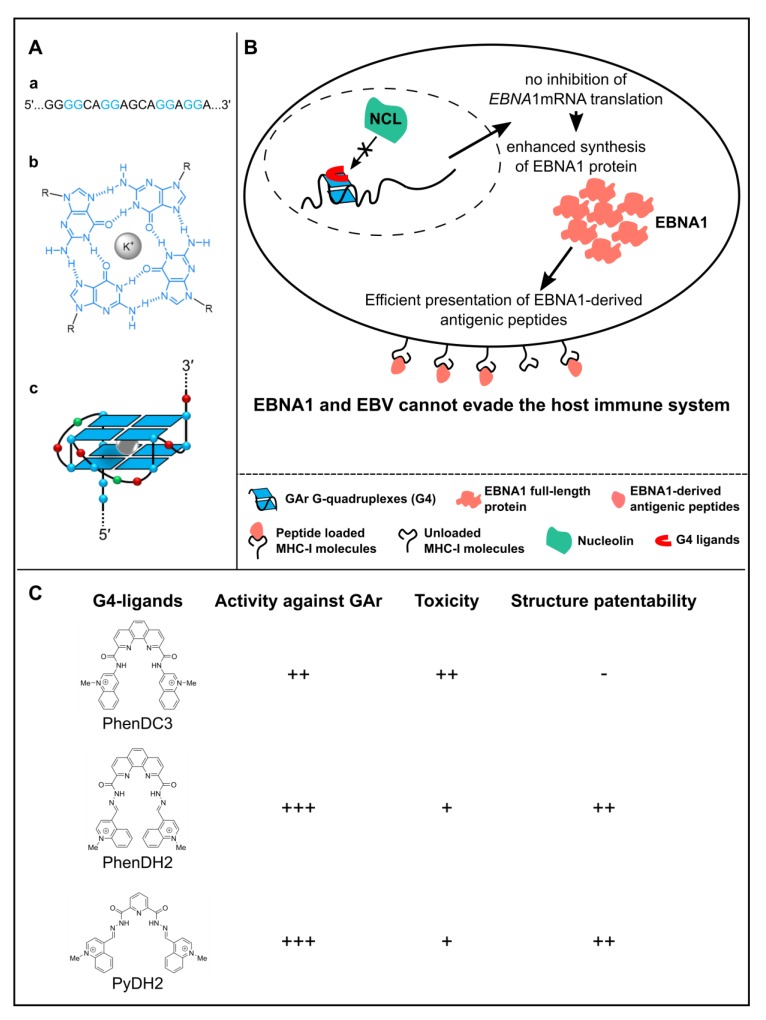
Model of nucleolin (NCL) role in EBNA1 immune evasion and of the mode of action of G4 ligands. (**A**) Structure of a G-quadruplex (G4). G4 are formed by the stacking of G-quartets that consists of a planar arrangement of four guanines connected by Hoogsteen hydrogen bonds and stabilized by a central cation, most often a K^+^. (a) Sequence of the repeated G4-forming motif within the GAr domain-encoding sequence of EBNA1 mRNA. (b) Structure of a G-quartet, formed by self-association of four guanine residues (blue) with metal cation (K^+^); R = ribose residue. (c) Schematic depiction of a putative two-quartet G4 structure formed in the GAr domain-encoding sequence of EBNA1 mRNA; nucleobase colors: G, blue; A, red; C, green. (**B**) Model of the role of NCL in EBNA1/EBV immune evasion and of the ability of some G4 ligands to interfere with this mechanism. The cellular NCL protein binds to G-quadruplex (G4) that form in the GAr-encoding sequence of the viral EBNA1 mRNA, thereby leading to inhibition of the translation of the latter, thus leading to a limited production of EBNA1-derived antigenic peptides and this way to immune evasion of EBV-infected cells. Some G4 ligands, such as PhenDC3, PhenDH2 or PyDH2, prevent the binding of NCL on G4 of EBNA1 mRNA, thereby interfering with the GAr ability to inhibit translation of its own mRNA, which results in an increase in the production of EBNA1 protein and of EBNA1-derived antigenic peptides. This may unveil EBV-infected cells to the immune system. (**C**) PhenDC3, which is active against GAr [41], does not display an original chemical structure as it is a benchmark G4 ligand. In contrast, PhenDH2 and PyDH2, which are less toxic and more active against GAr than PhenDC3, represent patentable compounds [54].

On the ground of these validations, a genetic screening was performed based on this yeast model for the mechanism at the basis of EBV immune evasion in latency. The rationale was to isolate host cell factors potentially involved in this mechanism and that could thus represent therapeutic targets to unveil EBV-related cancers, and more generally EBV-infected cells, to the immune system [17,41]. This way, nucleolin (Nsr1p in yeast, NCL in humans) was identified (Figure 2C, bottom) as the first host cell factor critically involved in EBNA1/EBV immune evasion. Indeed, in both the yeast model and human cells, overexpression of nucleolin exacerbated the inhibitory effect of GAr on translation whereas its down-regulation led to alleviation of this effect and of GAr-based limitation of antigen presentation by the MHC class I. Interestingly, at the time of this discovery, nucleolin was already known as the first G-quadruplex (G4)-binding protein identified [42,43] and G4 had been recently characterized in the GAr-encoding sequence of EBNA1 mRNA [44]. G4 are non-canonical secondary structures that may form in guanine-rich nucleic acids, both in DNA and RNA. G4 are formed by the stacking of G-quartets which consists of a planar arrangement of four guanines connected by Hoogsteen hydrogen bonds and stabilized by a central cation, most often a K^+^ (Figure 3A). G4 structures within G-rich DNA or RNA have been implicated in gene regulation where they can affect transcription, splicing or translation [45,46,47,48,49,50]. Hence, was tested the possibility that NCL binds to G4 that form in the GAr-encoding sequence of EBNA1 mRNA, thus leading to inhibition of its translation. Based on RNA pulldown [51] and proximity ligation assay (PLA) adapted to protein/RNA interaction [52], this model was validated (Figure 3B). Of note, this model fully explains the GAr length dependency of the *cis* inhibition of translation as the longer GAr is, the more it forms G4 (the full-length 235GAr being reported to form up to 13 G4 in vitro [44]), hence the more NCL proteins it could bind. In any event, this model also suggested that the NCL/G4 of EBNA1 mRNA interaction represents a *bona fide* intervention point to unveil EBV-related cancers to the immune system. This putative therapeutic target was validated since some G4 ligands, which include PhenDC3, by preventing binding of NCL to G4 of EBNA1 mRNA, interfere with the GAr-based inhibition of translation and limitation of antigen presentation by MHC class I (Figure 3B) [41]. Since PhenDC3 is a benchmark G4 ligand [53], new G4 ligands active in this system and with patentable structures have been investigated. This way, bis(acylhydrazones) PhenDH2 and PyDH2 (Figure 3C) have been developed and shown to interfere even more efficiently than PhenDC3 with the NCL/G4 of EBNA1 mRNA interaction and thereby with GAr-based limitation of protein expression and antigen presentation [54]. In addition, these two compounds present a lower toxicity than PhenDC3. Of note, the ability of G4 ligands to interfere with the NCL/G4 of EBNA1 mRNA interaction is not a general property of all G4 ligands as most novel PhenDC3 derivatives, despite strong binding and stabilization of G4-RNA in vitro, did not present this ability and thus were not able to interfere with EBNA1 stealthiness [54]. This is also true for pyridostatin (PDS), another benchmark G4 ligand, and probably due to a different mode of binding of all these compounds on G4 [41]. Förster resonance energy transfer (FRET)-based experiments on single RNA molecules may help to clarify and provide molecular bases to explain such a difference between various G4 ligands [55,56].

Hence, starting from a naive model based on budding yeast, which obviously has no common evolutionary history with EBV, a human virus, the first host cell factor involved in EBV immune evasion, NCL, has been identified and its mechanism of action deciphered [17,41]. Based on these findings, candidate drugs able to interfere with EBV immune evasion have been isolated [41,54]. Finally, the results obtained in yeast about the role of NCL binding on G4 of EBNA1 mRNA were at the basis of a study based on human cells and dedicated to the relationship between cellular compartmentalization and production of antigenic peptides that has suggested that nuclear processing of nascent transcripts determines synthesis of full-length proteins and antigenic peptides [57]. Indeed, this work indicates that antigenic peptides may derive from a nuclear non-canonical translation event that is independently regulated from the synthesis of full-length proteins and further show that G4 are exploited to control mRNA localization and translation by distinguishable mechanisms that are targets for viral immune evasion. This way, once again, budding yeast has been instrumental to model pathophysiological mechanisms and identify new therapeutic targets and candidate drugs. In line, G4 have been discovered and described in vitro for a long time by biophysicists but their functional role in various biological processes has emerged only relatively recently and, again, budding yeast played an important role in this expanding scientific field [58]. Notably, the role of G4 in genome stability and of the ability of some helicases to bind and unwind these particular secondary structures of DNA have been studied in detail in budding yeast. Indeed, repetitive DNA sequences, the most prominent and thus the most vulnerable ones being the rDNA repeats, telomeres, minisatellites and transposable elements, are a major threat to genome stability as they often drive chromosomes rearrangements therefore leading to various disorders. G4 can modulate the function of these repetitive DNA loci by affecting their transcription, replication and stability (for reviews, [58,59]). In particular, the Pif helicase family which play multiple roles in the maintenance of nuclear and mitochondrial DNA in eukaryotes has been extensively studied in budding yeast *S. cerevisiae* [60]. Hence, ScPif1 (*S. cerevisiae* Pif1) has been involved in replication through its ability to resolve barriers to replication such as G4, and thus reduces genetic instability at these sites [61,62,63,64,65].

## 4. Yeast Provided Key Insights into the Molecular Mechanisms of Gene Repression and Switching at the Basis of Immune Evasion of Some Parasites

The ability of many parasites, which includes many pathogenic protozoan and fungi such as *Plasmodium*, *Trypanosoma*, *Candida*, or *Pneumocystis*, to evade the host immune system is key to their success and pervasiveness. Their genomes contain large family of genes encoding closely related surface proteins (e.g., *VAR* genes in *Plasmodium* [66,67,68,69], variant surface glycoprotein (*VSG*) genes in *Trypanosoma* [70], major surface glycoprotein (*MSG*) genes in *Pneumocystis* [71,72]) and exquisite regulatory mechanisms have been developed so that, at any time, only one of these genes is expressed due to efficient mechanisms that silence all the other genes of the family. As these surface proteins are immunogenic, rare switching to other members of the gene family favor the immune evasion of the pathogen but, for most of them, both the mechanisms allowing gene silencing and switching are poorly understood. However, studies in the budding yeast *S. cerevisiae* have revealed similar mechanisms of gene repression and switching, which involve either epigenetic switching or recombination, and have therefore provided significant insights into the molecular bases of these phenomena [73]. Briefly, gene silencing is mediated by compact heterochromatin formation that is re-established after cell division. Of note, the aforementioned families of genes are located mostly or exclusively in the subtelomeric regions of the chromosomes [73], whereas some of the very few located elsewhere are surrounded by telomeric repeats [74]. Telomeres are long-known sites of gene silencing and this telomere position effect (TPE) has been extensively studied in *S. cerevisiae* [75]. Importantly, unlike many higher eukaryotes, DNA methylation and RNAi-based mechanisms do not play a role in the repression of subtelomeric genes in budding yeast. Hence, due to these particularities, one may consider *S. cerevisiae* an irrelevant model organism for epigenetics in higher eukaryote. However, these differences make budding yeast quite relevant for modelling the antigenic variation of parasites discussed here, as DNA methylation and RNAi do not seem to contribute to the silencing of the variance genes [68,75]. Also, one of the key questions when it comes to selectively express only one gene of an extended gene family is the mechanism that allows such a selectivity and clues again came from budding yeast *S. cerevisiae* where clear relationship exists between gene expression and gene position in the nucleus, in particular for telomeric and subtelomeric sequences [76]. Indeed, in budding yeast, due to a handful of proteins, which include Ku and some nuclear pore components, the telomeres cluster in several compartments at the nuclear periphery where genes are silenced and translocation of telomeres inside the nucleoplasm is associated with loss of repression of the telomeric genes [76,77]. Interestingly, a similar clustering of the inactive *VSG* genes in *Trypanosoma* or of the inactive *VAR* genes in *Plasmodium* has been observed, and the active VAR genes leave these clusters. However, they remain quite close to the repressed cluster, and still at the nuclear periphery, suggesting a slightly different regulatory mechanism [68,69]. Hence, the study of telomeric gene silencing have provided a basic framework for the understanding of the silencing of and epigenetic switches within extended families of genes that encode major parasite antigens. Another important mechanism to switch from one member of the family to another is DNA recombination. This particular mechanism has been extensively studied in budding yeast as it is involved in the mating-type switching [78], a process that ensures that both haploid types (Mata and Matα) persist in order to allow mating, as, in the wild, *S. cerevisiae* preferentially grows as a diploid. In this case, a similar constitutive repression of donor genes exists that, to be expressed, need to be translocated to an active site by a DNA recombination event that is favored by the homothallic switching endonuclease (HO), thereby leading to mating-type switching. Here again, budding yeast *S. cerevisiae* provided a model mechanism for the role of DNA recombination which seems especially important for the epigenetic switching within *MSG* genes in *Pneumocystis* in which the expression site also determines the expressed variant [71,72]. 

## 5. Yeast as a Pathogen: Its Own Strategy to Evade the Immune System

**Figure 4:**
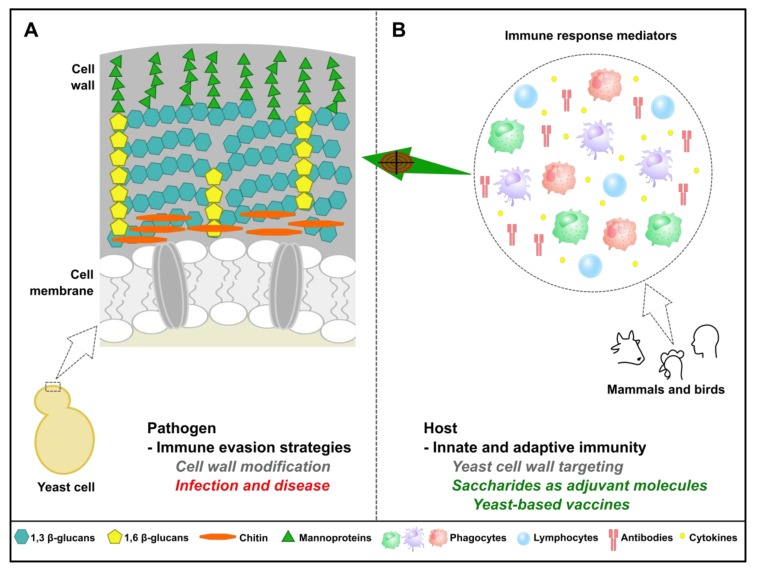
Importance of yeast cell wall components in immune detection and vaccine development. (**A**) Yeast has evolved a sophisticated cell wall enabling better adaptation to harsh environmental conditions. Pathogenic fungi can further modify cell wall components to resist host immune response pressure and cause disease. (**B**) On the other hand, carbohydrates abundantly found in yeast cell wall are absent in mammals and poultry birds, enabling efficient recognition of yeast cells by the host immune surveillance mechanisms, in particular by the innate immune system. This particularity has been exploited for the development of new adjuvant molecules and yeast-based approaches as alternatives to alum-adjuvanted subunit vaccines.

Yeast can also be a pathogen for humans or other animals and fungi such as *Candida albicans* (which is genetically and morphologically very close to *S. cerevisiae*) or from the *Aspergillus* genus represent a major threat for public health, especially in immune-compromised individuals. The fungal cell wall, which plays an essential role in fungal morphogenesis and resistance to osmotic shock, represents around 30% of the yeast cell dry weight, up to 50% of the cell volume and polysaccharides represent over 90% of its components. These polysaccharides are the molecular scaffold that supports cell wall proteins, lipids and superficial components [79,80]. The cell wall crucial and active role during fungal infection, in humans and other animals, has been recognized for a long time (Figure 4). Indeed, the first step in the development of an immunological response against pathogenic fungi involves recognition of some component of their cell wall by phagocytic cells from the innate immune system. Innate immune cells exploit a range of Toll-like receptors (TLRs, which include TLR-4 and TLR-2) as well as C-type lectin receptors (CLRs) such as Dectin-1, DC-sign, and mannose receptors, to detect the so-called pathogen-associated molecular patterns (PAMPs), many of which in fungi are components of the cell wall that include β-glucans, mannans, mannoproteins and chitin [81,82]. In this respect, some cell wall components, in particular β-1,3-glucan, can be considered to be *bona fide* Achilles heels of pathogenic fungi as they are essential, or at least of extreme importance, for fungi survival and, at the same time, highly immunogenic. Consequently, changes in PAMP exposure at the fungal cell surface affect immune recognition, thereby influencing yeast ability to colonize its host. Hence, among various strategies to evade the immune system, changing cell wall composition or masking some highly antigenic cell wall components have been developed by various pathogenic fungi. For example, some fungi have developed a strategy based on covering β-1,3-glucan and chitin by various molecules that include mannans [83,84,85,86,87,88,89,90] or α-1,3-glucan [91], thereby affecting its recognition by the Dectin-1 receptor. Another strategy developed by the yeast *Histoplasma capsulatum* is to secrete Eng1, an endo- β-1,3-glucanase which, by trimming β-1,3-glucan fragments exposed at the fungal cell surface, minimize their recognition by Dectin-1 [92]. 

Interestingly, this cell-surface epitope-masking can be induced by alterations in the environment. Hence *C. albicans* reacts to the change in carbon source from glucose, which is mostly absent in vivo, to lactate, which is the major carbon source in the blood or in vaginal environment, by masking β-glucan thereby protecting itself from the host immune response [93,94]. This last case is a nice illustration of the so-called adaptive prediction or anticipatory response in which certain pathogens, including pathogenic yeasts from the *Candida*, *Aspergillus*, or *Histoplasma* genera, have evolved anticipatory behaviors that are triggered by specific signals in the host to prepare themselves against imminent host challenges. This was nicely reviewed recently [95] and has been proposed to represent a primitive form of microbial memory which would somehow be reminiscent of the anticipatory response of Pavlov’s dogs. In the case of *C. albicans*, this pathogenic yeast is prepared to host-specific conditions such as high lactate and hypoxia and developed an anticipatory response that leads to epitope-masking once inside its host. Interestingly, this type of anticipatory response seems to have been developed preferentially in pathogenic fungi such as *C. albicans* that is likely to have evolved in the mammalian host, whereas the benign budding yeast *S. cerevisiae*, which does not have the same evolutionary history, does not necessarily display the same mechanisms. As an illustration is the fact that glucose represses stress resistance in *S. cerevisiae* [96,97], whereas it leads to activation of oxidative stress genes in *C. albicans* [98]. As a direct consequence, glucose protects *C. albicans* against killing by circulatory neutrophils in the bloodstream where the concentration of glucose is relatively high [99].

## 6. Biotechnological Exploitation of Yeast to Create Vaccine or Adjuvant for Human and Animal Vaccination

As stated above, yeast-based approaches have opened unique windows of opportunities to decipher eukaryotic cell biology and to produce molecules of biotechnological relevance. More recently, these microorganisms have also found a special place in the vaccinology field. The observation that host immune response against fungi is robust and evolutionarily conserved in the animal kingdom raised the question whether yeasts can be employed as platforms for the development of novel prophylactic and therapeutic strategies against cancer and infectious diseases. 

Use of yeast as workhorses for the production of vaccines is an appealing idea for different reasons. Despite the immune evasion mechanisms aforementioned, innate recognition of fungi leads to strong adaptive immunity and if host defenses are intact, fungal infections are very rare [100,101]. Moreover, yeasts have been largely used as probiotics in human and veterinary medicine for preventing infections by trapping pathogenic bacteria, modifying myeloid cells phenotype, migration and T-cell polarization capacity [102]. These particularities have been associated with the presence of conserved yeast cell wall carbohydrates that are absent in hosts including mammals and poultry birds and hence are easily targeted by immune surveillance mechanisms [103,104,105]. Due to their immunogenicity and capacity to modulate immune response, these carbohydrates have great potential as novel adjuvants molecules. Yeast cell wall comprises an inner layer of β-1,3-glucan chains (80–90%), β-1,6-glucan (8–18%) and chitin (1–2%) and an outer layer of mannoproteins (Figure 4) [106]. The inner layer is responsible for the mechanical strength and elasticity of cell wall whereas the most external layer of the cell wall is involved in several types of interactions between fungal cells and host immune system [107,108]. 

β-Glucans enhance resistance to bacterial and parasite infections and play a role in protective immunity. These molecules are recognized by dectin-1 and complement receptor 3 (CR3) resulting in fungal opsonization, inflammatory cells influx, Th17, Th1 and cytotoxic T-cell responses [109,110,111]. For this reason, β-glucans are promising alternatives to surpass the Th2-type (antibody)-biased responses elicited by alum-adjuvanted vaccines and have been exploited in clinical conditions requiring the induction of T cells defenses such as cancer [109,110,111,112]. β-Glucans from different sources are easily obtainable and their derivatives have been extensively developed into vaccine adjuvants or vaccine delivery systems [113]. However, other yeast polysaccharides, such as mannan and chitin, can also bind pattern recognition receptors (PRR) and stimulate the immune system [114]. Glucan-mannan particles (GMP), glucan-chitin particles (GCP) and glucan-chitin-mannan particles (GCMP) have been generated and showed to be safe in both preclinical and human trials. Pure-glucan particles and GCP promote comparable levels of Th1 response but GCP have been reported as better inducers of Th17 immunity. These differences are attributed to the higher levels of chitin found in GCP and highlight the potential of this polysaccharide as a tool for boosting Th17-type immune response [115]. Yeast mutant strains carrying higher amounts of chitin increase activation of macrophages and show reduced colonization capacity and virulence [116]. Moreover, chitosan microparticles derived from chitin increase mucosal and systemic immune responses by improving cell permeability and antigen stability [117]. Whereas mammalian proteins rarely have exposed mannose residues, fungi use mannose as their preferred sugar [118]. Interestingly, glycosylated proteins from yeast cell wall trigger immune mechanisms such as inflammasome activation and pyroptosis [119] and fungi lacking β-1,3-glucan shed a massive amount of galactomannan [120]. Mannans are highly mannosylated polysaccharides exposed at the most external layer of yeast cell wall that interact with dendritic cells (DC) mannose receptor, mannan-binding protein, dectin-2 and DC-specific intercellular adhesion molecule 3 (DC-SIGN). Mannan polysaccharides induce the release of pro-inflammatory cytokines, DC maturation [108] and induce better Th1-Th17 signature on inflammatory DC than β-1,3-glucan [120]. Yeast mannan-conjugates give promising results in allergen-specific immunotherapy by enhancing allergen bioavailability and uptake by DC [121]. In addition, these conjugates are described as efficient platforms for the development of antifungal vaccines [122].

Development of new vaccine compositions has been generally based on subunit modalities due to stringent regulatory and safety issues. In these formulations, pathogen antigenic components are administered with strong adjuvants that have little capacity to induce cellular response and can provoke aberrant local and systemic reactogenicity in susceptible individuals [123]. Oral vaccines have attracted a great deal of attention as they enable a higher capacity for mass immunizations during pandemics at relatively low cost and confer enhanced mucosal and systemic immune responses [124]. Development of new vaccines for farm animals is further driven by cost-effective alternatives that do not need to be administered individually and can be stored without a cooling chain [125]. In this context, the inherent immunogenic nature of yeasts combined with their versatility as heterologous protein expression systems and their probiotic activity might provide innovative strategies to fight human and animal diseases for which effective vaccines are lacking. Novel ways of employing yeast against infectious diseases and cancer include the use of virus-like particles (VLP), whole recombinant yeast (WRY) and yeast surface display (YSD). 

Subunit vaccines based on soluble, monomeric proteins often present limitations regarding immunogenicity that can be overcome by VLP-based approaches [126]. VLP are multimeric complexes of virus proteins produced by recombinant DNA technology. They have received much attention as vaccine candidates because they have antigenicity and immunogenicity similar to native virions but are non-infectious and lack viral genomic material [127,128]. Foreign antigens can be displayed on VLP surface by genetic engineering or by chemical conjugation. These particles are suitable for mucosal immunization due to their resistance to proteolytic degradation and compatibility with mucosal environment [129]. Yeast VLP scaffolds have been used to display immunogenic antigens from pathogens such as porcine circovirus [130], rabbit hemorrhagic disease virus [131], coxsackievirus A6 [132], hepatitis B virus [133], Zika virus [128], chikungunya virus [134], dengue virus [135], enterovirus [127,136]. 

Unlike peptide-based vaccines, WRY or YSD-based vaccines do not require special adjuvant [137]. Yeasts are non-toxic and easy-to-grow microorganisms, some species being able to multiply at physiological temperatures and survive the harsh environmental conditions found in the gastrointestinal tract. Moreover, yeast cannot acquire or spread genes conferring resistance to antibiotics and are consequently safer than probiotic bacteria [138]. Recombinant yeasts represent then interesting tools for developing edible vaccines for human and animals. WRY has attracted high attention for preventive and therapeutic vaccine formulations against several chronic infections and cancers. In this approach, yeast cells are used as both production and delivery systems, providing a simple and affordable vaccine production platform free from complicated and expensive downstream protein purification steps [112,139]. Kim et al. [129] observed that oral administration of WRY provoked 9–27 times higher antibody titers than purified antigen. Interestingly, both heat-killed and live yeast generate antigen-specific immune response and elicit equivalent protective immunity, circumventing the risk of pathogenicity of live vaccines [112]. Tarmogens (targeted molecular immunogen) use intact heat-inactivated *S. cerevisiae* cells as vectors to elicit antigen-specific CD4+ and CD8+ T-cell responses. This approach has been successfully employed to treat patients with chronic hepatitis C virus infection [140] but did not result in a clinical benefit in patients with chronic hepatitis B [141]. In preclinical studies, another Tarmogen-based vaccine promoted minimal prophylactic activity against *Mycobacterium tuberculosis* but reduced lung pathology and improved survival rates in a therapeutic model of tuberculosis [142]. Other WRY showed to elicit cell-mediated and/or humoral immune responses against dengue virus [124], fish nervous necrosis virus [129], porcine epidemic diarrhea virus [143], infectious bursal disease of poultry [144] and malaria [145]. 

WRY are also in development for cancers therapies. Yeast expressing human carcinoembryonic antigen (CEA) has been engineered to circumvent the non-immunogenic nature of cancer-associated antigens and induced killing of human CEA+ tumor cells in preclinical studies [146,147,148]. WRY expressing brachyury, a nuclear transcription factor expressed in a range of human carcinomas and chordoma, has been tested in a phase I clinical study that demonstrates the safety and efficiency of this therapeutic cancer vaccine. A randomized phase II chordoma study employing this vaccine is currently in progress [149]. WRY induced antitumor immunity and improved survival rates in a murine model of melanoma [150,151], and heat-inactivated whole yeast proved to be a better delivery system than killed recombinant *Leishmania* in efforts to prevent high-risk HPV [152]. 

Cell-surface display has been widely used to optimize WRY by improving antigen targeting, giving rise to YSD. In this approach, yeast cells are transformed with a single vector encoding a protein variant of interest that is genetically fused to a cell-surface anchor protein. The anchor protein contains a signal sequence that directs efficient transport of the fusion protein to the cell surface, where it is immobilized and accessible to the extracellular space [153]. YSD has been mostly used in oral vaccines for animal diseases including chicken coccidiosis [154], porcine pleuropneumonia [155] and porcine circovirus associated disease [125], grass carp hemorrhagic disease [156] and fish infectious hematopoietic necrosis [157]. Of note, in addition to stimulate humoral and cell-mediated immune responses, oral administration of YSD has a positive impact on animal health through its direct nutritional effect [154]. Palma et al. [158] observed that probiotic YSD expressing HIV-1 gag protein induce CD8+ T-cell response in HIV-1+ individuals, highlighting genetically engineered probiotic *S. cerevisiae* strains as promising vectors for HIV vaccines. YSD has been also reported as a technology allowing rapid and large-scale production of influenza vaccines [159]. Comprehensive lists of yeast-based vaccines for human and animal diseases are described elsewhere [112,127,137].

The latest advances in vaccinology have had a major effect on public and animal health. However, efficient vaccines targeting important pathogens and cancer remain an ongoing challenge. Vaccine-mediated protection might work best if the response is skewed towards a particular mechanism such as cytotoxic, Th1-, or Th17-type responses and methods enabling this modulation are still poorly developed. In this context, yeast adjuvant molecules and yeast-based vaccines hold great value in human and veterinary medicine and might open new avenues for controlling and treating infectious diseases and malignancies.

## 7. Concluding Remarks and Perspectives

Another use of yeast as a biotechnological tool is the production of recombinant EBV protein for use as an EBV vaccine. Indeed, EBV is recognized as an important vaccine target, in particular for cancer prevention and several efforts has been undertaken, most of them being focused on the EBV glycoprotein gp350 which is the most abundant glycoprotein on the virus and on virus-infected cells and the main target of naturally occurring neutralizing antibodies [23,160]. This way the yeast *Pichia pastoris*, which is a popular workhorse for production of recombinant proteins, has been exploited to express truncated forms of EBNA1 or gp350, both as secretory proteins with an N-terminal histidine tag that allow their purification [161,162]. In both cases, the truncated EBV proteins retained good immunogenicity and could therefore represent a useful source for developing EBV vaccine candidates. Importantly these studies highlight that expression of various immunogenic EBV proteins in yeast does not significantly alter their immunogenicity, which is consistent with the successful use of yeast in general, and *P. pastoris* in particular, for the development of subunit vaccines against a wide range of diseases caused by bacteria and viruses. In addition, *S. cerevisiae* has been used in the manufacture of a dozen of approved vaccines against hepatitis B virus and one against human papillomavirus [163]. Hence, one can imagine to combine the expression of several EBV protein (e.g., EBNA1 and gp350) as membrane associated proteins in the same yeast strain and to use these cells as WRY or YSD vaccines, with the additional interest that they do not require special adjuvant as the yeast cell wall itself represents an efficient adjuvant.

Another important point is that the aforementioned use of budding yeast to model the mechanism at the basis of the immune evasion of EBV could be extended to other oncoviruses. For example, similarly to EBV, the immune evasion of the oncogenic Kaposi sarcoma-associated virus (KSHV) involves its genome maintenance protein LANA-1 which is also able to self-limit the translation of its own mRNA [164] via a mechanism that, as for the EBNA1 protein of EBV, is probably based on G4 within the coding sequence of its own mRNA [44]. Hence, the same type of yeast-based approach than the one developed for EBNA1 could be envisioned for other human oncovirus.

Hence, this review article dedicated to the link between yeast and immune response and evasion nicely illustrates all the possible uses of yeast, from a popular model system to decipher basic cellular regulatory pathways as well as pathophysiological mechanisms to a convenient and versatile tool for chemical biology and for biotechnological and pharmacological applications [165], not to mention the historical and essential use of yeast as a leavening agent and for alcoholic fermentation: now it is time for a beer!
